# Experience with radiopharmacy information system

**DOI:** 10.1186/s41181-022-00169-w

**Published:** 2022-07-19

**Authors:** Jesús Luis Gómez Perales

**Affiliations:** Nuclear Medicine Service, Puerta del Mar University Hospital, Avenida Ana de Viya 21, 11009 Cádiz, Spain

**Keywords:** Radiopharmacy management, Nuclear medicine, Radiopharmaceuticals traceability, Database software

## Abstract

**Background:**

The efficient management of hospital radiopharmacy is very important for a good workflow in nuclear medicine and essential to ensure the correct traceability of the radiopharmaceuticals administered to patients. In the hospital radiopharmacy of our nuclear medicine department, it was developed and implemented a radiopharmacy information system (Radiolab).

**Results:**

From its implementation, important additional functionalities have been added to this software application, according to our requirements and according as well to some of the specific requirements proposed in a recently published consensual tool for validating radiopharmacy software. After more than ten years from the implementation of this software application, it has been analysed its contribution to our hospital radiopharmacy and nuclear medicine department.

**Conclusions:**

As a result, this radiopharmacy information system provides comprehensive management of our hospital radiopharmacy in an efficient way, and reliable traceability of every dose of radiopharmaceutical administered in our nuclear medicine department.

## Introduction

Hospital radiopharmacies are facilities for small-scale preparation and dispensation of radiopharmaceuticals, usually located within or very close to a nuclear medicine service. A nuclear medicine information system (NMIS) is a software application for the management of nuclear medicine departments (NMD). And a radiopharmacy information system (RPIS) is a software application for the management of hospital radiopharmacies. In our NMD, we had both a NMIS and a RPIS from an external supplying company. But, many years ago, our NMD merged with the radiology department of our hospital to configure a unified diagnostic imaging department. In this context, we had to replace our NMIS and RPIS for a radiological information system (RIS). Although a few years later we separate again into two independent departments, we still must keep using that RIS to manage our NMD.

RIS is the core system for the electronic management of imaging departments. It complements hospital information system (HIS) and picture archiving and communication system (PACS) (Kinsey et al. 2000) and it is critical to efficient workflow into a radiology department. So, RIS is basically evolved for the needs of radiologists and mainly used to schedule patient appointments, and to manipulate or to distribute radiographic images and data. However, the workflow in a NMD is different from that of a radiology department. Therefore, a NMIS expands the functionalities of RIS to include additional functions, which are essential for an efficient workflow in any NMD (Volkan-Salanci et al. 2012). However, on the other hand, a NMIS cannot cover the entire spectrum of functionalities needed for the proper functioning of a hospital radiopharmacy and, therefore, it cannot guarantee complete and reliable traceability. The radiopharmaceutical health products circuit is a complex process that requires computerization (Blondeel-Gomes et al. 2020), and in this sense all the work and functions carried out in a hospital radiopharmacy have more than enough entity to require its own software information system, to guarantee adequate and efficient management as well as full traceability.

RIS does not record important data, such as the activities of the radiopharmaceutical doses administered to the patients, the batches of the radiopharmaceuticals, etc. And NMIS does not record the information about important functions carried out in a hospital radiopharmacy, namely, orders, stocks, elutions and disposal of generators, the different types of radiopharmaceutical preparations (labellings), quality controls, doses dispensing and radioactive waste. All of which are strictly necessary to ensure efficient management of hospital radiopharmacies and correct traceability of the radiopharmaceuticals administered in NMD.

Faced with this problem, I tried to find a solution already available in the market, that is, some commercial software application. But things got tricky because different problems arose: in some cases, the software was focused on centralized radiopharmacies, in other cases, the software was a web application working on a distant server and accessed by a browser, and in other cases, the problem was simply that the program was too expensive. Furthermore, I couldn’t find any of them that exactly fit the needs of my radiopharmacy. So, I decided to develop and implement our own RPIS, and I called it Radiolab (Gómez-Perales 2013). Besides, I tried to make it as flexible as possible as well, that is, being easy to adapt to the needs of any radiopharmacy.

After ten years from its implementation, it is analysed the contribution of this RPIS to our hospital radiopharmacy and nuclear medicine department.

## Materials and methods

The development process of this RPIS included several basic stages: specifications, design, implementation, testing, deployment and maintenance (Rosen 1998). Its database (back-end) was developed in MS Access, and the language used for programming the front-end of the software is Visual Basic for Applications (VBA). After been implemented in our NMD, its features and functionalities were already described (Gómez-Perales 2013). Basically, it has twelve modules whit the following functionalities:

- Orders: orders management of radiopharmaceuticals, cold kits and disposable materials.
- Stock: inventories of radioactive products, cold kits and disposable materials.
- Generators: management of elutions and disposal of ^99^Mo/^99m^Tc generators. It also calculates the elution efficiency of each eluate.
- Labelling: management of labelling of cold kits, leukocytes, red blood cells and platelets.
- Controls: for recording all data related with tests necessary to ensure compliance with established specifications and standards, including examinations and assays (activimeters, radiochemical purity, cleaning, microbiological, temperature and radiochromatograph).
- Dispensing: for prescriptions import from the NMIS or RIS to the RPIS, and for management of radiopharmaceuticals dispensing.
- Waste: management of the radioactive waste and their disposal.
- Traceability: it provides complete traceability of all the radiopharmaceuticals administered to the patients.
- Protocols: it links to the instructions and approved standard operating procedures for each procedure or operation within the radiopharmacy, such as preparations and quality control activities.
- Reports: for generating and managing all kind of reports, such as incidents, complaints, inspections, etc.
- Maintenance: it performs the customization processes of the RPIS and several tasks for the database maintenance, such as the backup or compaction of the database.
- Agenda: an address book and a screen for managing information related to tasks of the radiopharmacy.

This RPIS has user and password access control, and it can work both as a single-user desktop application and as a multiuser network system. The database of this RPIS is in the server of our hospital network, and we connect to it from several computers in our NMD and our hospital radiopharmacy.

**Fig. 1:**
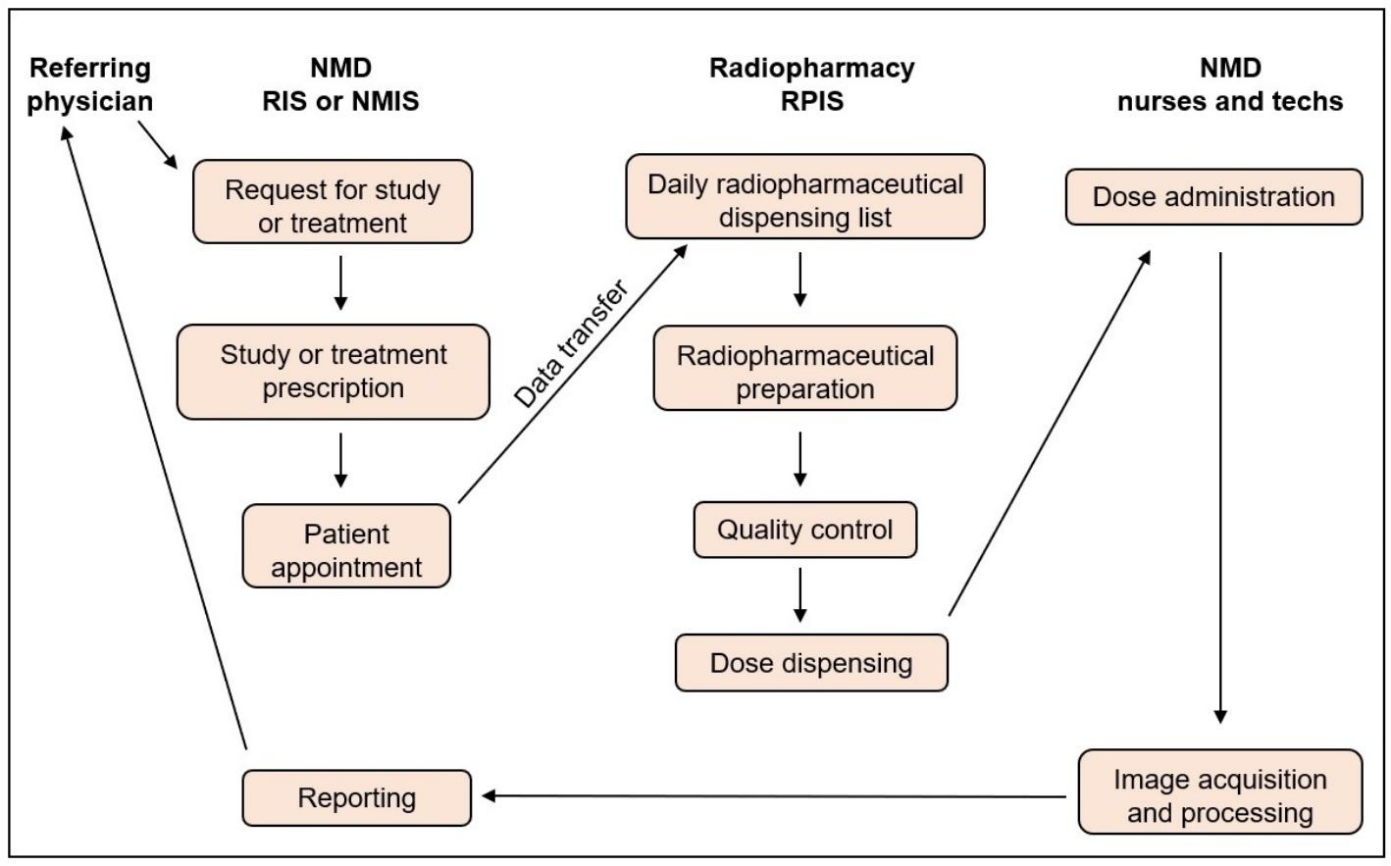
Simplified workflow scheme of our nuclear medicine department (NMD)

The workflow in our NMD is basically the following (Fig. 1). When the referring physicians request nuclear medicine tests for their patients via HIS, those requests are transferred to the RIS. The indication for every diagnostic study or treatment is validated by a nuclear medicine specialist. Then, the appointments are scheduled and the instructions for patient preparation are given to the patient through the nuclear medicine secretary or nursery staff. The day before the appointment, the data of all the radiopharmaceutical prescriptions are transferred from the RIS to the RPIS via an intermediate XLS or XLSX file. Specifically, the transferred data are: name of the patient, patient code, appointment date, appointment time, description of the study or treatment, radiopharmaceutical code, and the requesting or petitioner unit.

With those data, the RPIS issues a worksheet with the following data: date, dispensing times, patient names, radiopharmaceuticals and dose activity. The only exception is the emergency procedures.

Since the activity of the dose is not contemplated by the RIS, our RPIS assigns an activity to each radiopharmaceutical based on the radiopharmaceutical, the study or treatment, and the patient’s age. If the patient is over sixteen, the RPIS assigns the standard dose for adults, which is established in the Catalog of the Maintenance module. And if the patient is under sixteen, the RPIS can calculate the dose activity according to the EANM paediatric dosage card.

Once the radiopharmaceutical has been dispensed an administrated, and after the necessary time has elapsed in each case, the diagnostic images are acquired and processed. And finally, a nuclear medicine specialist makes the corresponding report.

## Results

We have been using this RPIS in our NMD for more than ten years. According to our requirements, from 2011 until now, the most outstanding functionalities added to the software are:

- Calculation of radioactive decay of radionuclides.
- Managing of ^68^Ge/^68^Ga generators.
- Instant prediction of theoretical activity available on every generator in stock.
- Assistant for labelling of cold kits.
- Calculation of the remaining activity into every radiopharmaceutical’s vials.
- Effective dose and cumulative effective dose received by each patient from all the radiopharmaceuticals administered, calculated according the International Commission on Radiological Protection (ICRP).
- Control of expenses in the Orders module.
- Control of expenses in the Dispensing module (billing reports).
- Control and traceability of users traffic entering and leaving the RPIS.
- Any user can make a backup of the Radiolab database at any time and save it wherever they want. In addition, every time a user closes the program, a backup of the Radiolab database is automatically saved on the computer from which it was closed.
- Radiolab’s database is protected by a password as complex as you like.

Furthermore, to improve the RPIS it has also been followed some of the specific requirements of the first published consensual tool for validating radiopharmacy software (Léa et al. 2020), proposed by a panel of experts of the French Society of Radiopharmacy (SoFRa), following the Delphi method (McPherson et al. 2018). According to this, the most outstanding functionalities added to the RPIS are:

- Manual in paper and electronic format.
- Trial software and procedure to test the software prior to production.
- Restriction access to the system based on responsibilities.
- Levels of access allocated according to each person’s authorization.
- Only the administrator can create users, change user rights and reset user’s passwords.
- Records and traceability of the creation, modification and removal of access rights.
- Alert of non-compliance between a radiopharmaceutical order or receipt and the nuclear safety authority authorization.
- Alert of non-conformity of a quality control for a radiopharmaceutical or a preparation.
- Alert of exceeding the prescribed activity in relation to the protocol threshold.
- Alert of expiration date less than a defined value.
- Alert of reaching a minimum threshold stock for a kit of radiopharmaceutical.
- Generation of labels with barcodes for radiopharmaceutical preparations, eluates, syringes and waste.
- Reports and system data can be converted into common formats, namely, MS Word, MS Excel, and PDF.

This RPIS allows statistical analysis of our NMD through a wide variety of reports. But also, since system data of this RPIS can also be converted into MS Excel format, it can be used the wide range of statistical functions of MS Excel.

**Fig. 2:**
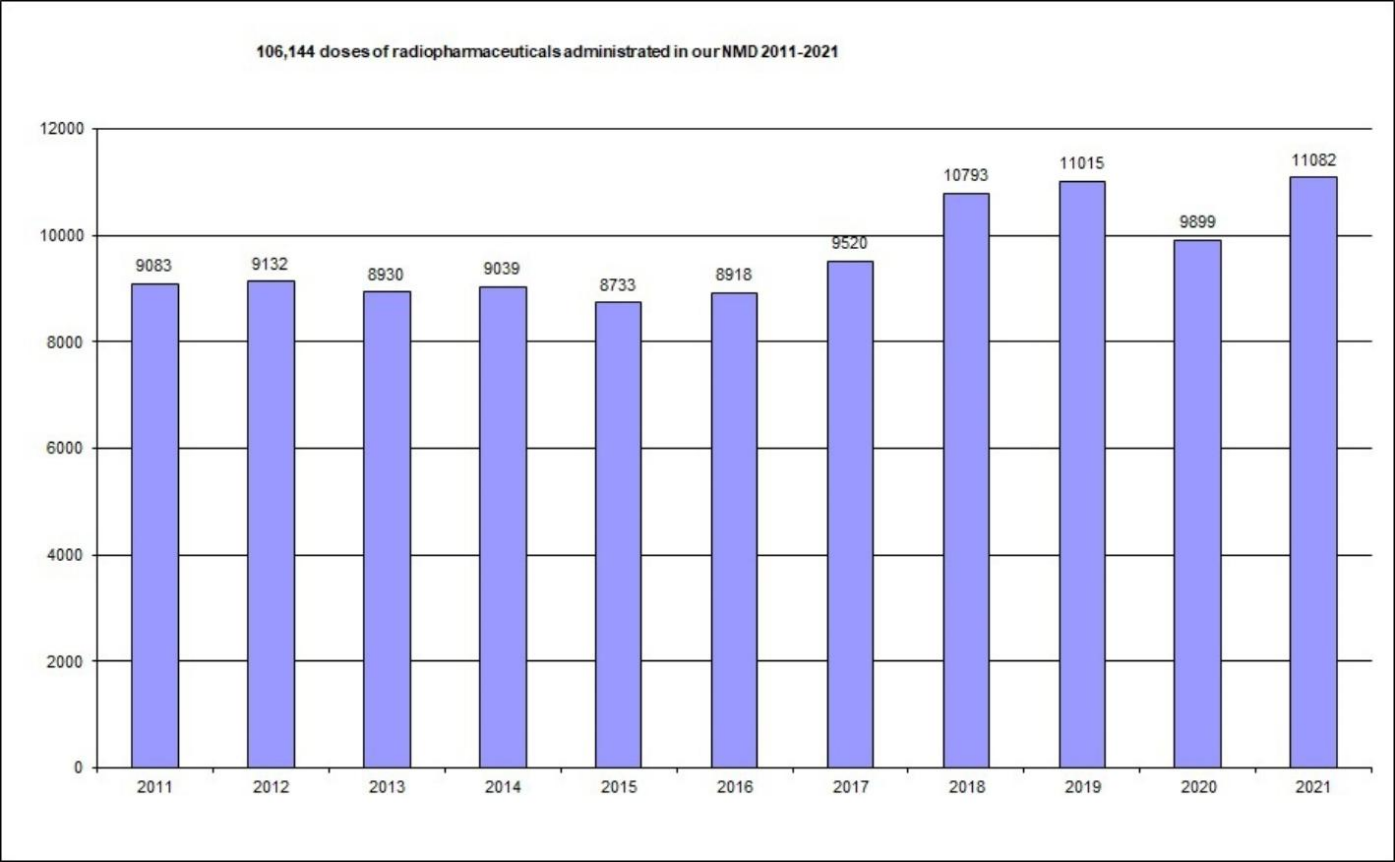
Distribution of radiopharmaceutical doses administrated from 2011 to 2021

**Fig. 3:**
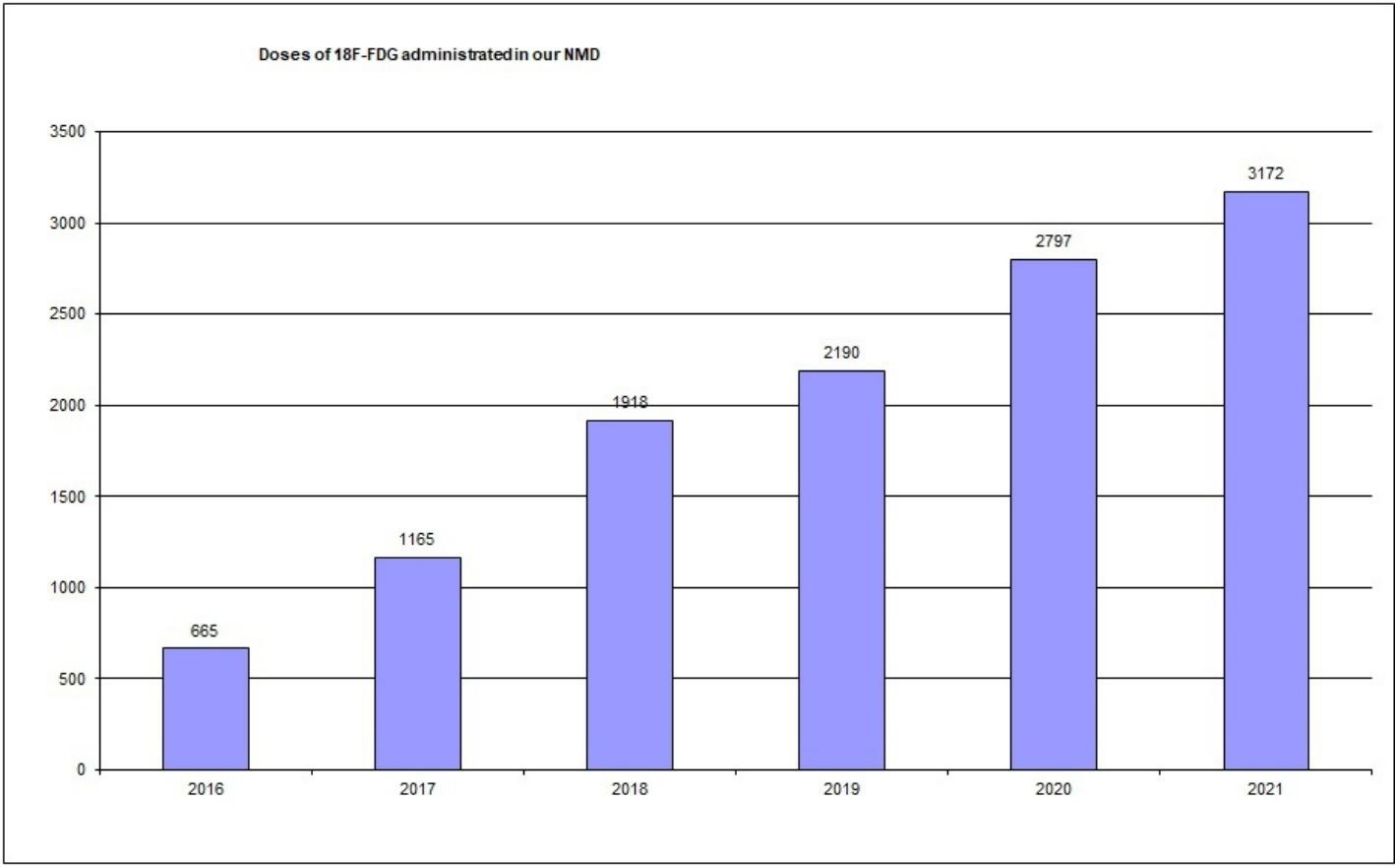
Doses of 18 F-FDG administrated from 2016 to 2021

So, reviewing performance statistics with our RPIS, we can quickly and easily access a lot of information. For example, between January 2011 and December 2021 (11 years), sixteen different radionuclides and fourteen different cold kits have been used for imaging or therapy, and nine different radionuclide therapies are given to patients in our NMD. A total of 106,144 radiopharmaceutical doses have been administrated (Fig. 2) (about 9,650 per year). A total of 16,098 kits have been labelled in our radiopharmacy (about 1,463 per year). A total of 23,144 radiochromatograms for radiochemical quality control have been performed (about 2,100 per year). We started performing PET studies with ^18^ F-FDG in 2016. Since then, the number of these studies has grown continuously (Fig. 3).

The Orders and the Stock modules have been a great help to keep the needs of our NMD perfectly covered in a very efficient way.

This RPIS offers perfect traceability of all our radiopharmaceutical preparations, dispensing and radioactive waste.

The size of the front-end of the RPIS is always the same (50 MB). But the size of the back-end has been growing, from an initial size of 2 MB to 59 MB ten years later.

We perform the import of the prescriptions data from RIS to RPIS twice for each date. Firstly, the day before the date of the prescriptions to elaborate the daily worksheet with all the scheduled prescriptions. And secondly, afterwards to import the not scheduled prescriptions, that is, those of the emergency procedures.

In addition, once the list with the daily prescriptions is imported from RIS to RPIS, it is necessary to review it to eliminate those that do not apply. For instance, the prescription in RIS of diagnostic studies with some radiopharmaceuticals (^67^Ga-citrate, SeHCAT, ^123^I-MIBG, etc.) appear twice, the first one for administration and the second one for scintigraphy or SPECT. Obviously, the first one must appear in the radiopharmacy’s worksheet, while the second one must be removed from it.

## Discussion

A NMIS can try to integrate radiopharmaceutical management functions, such as radiopharmaceutical preparation and dispensing. But the complexity of comprehensive radiopharmacy management is large enough to require an exclusive software application, that is, a RPIS. It is necessary to manage a great deal of processes and tasks, namely, management of orders and stocks of radipharmaceuticals, cold kits and disposable materials, elutions and disposal of generators, labelling of cold kits and autologous radiopharmaceuticals, quality controls, doses dispensing, management of radioactive waste, etc. The correct management of all those data make possible a correct traceability of all radiopharmaceutical products and their waste, which is of utmost importance.

Our RPIS (Radiolab) fulfils all these functions in a quite flexible way, that is, easy to adapt to the needs of any radiopharmacy. Because, let’s face it, although all radiopharmacies perform basically the same processes, the rate and extent of interpretation and adoption of international directives, such as cGRPP ( Gillings et al. 2021), varies among each country, and may introduce changes, provided the general scope and limits of each directive are maintained. Besides, even within the same country we can find differences in this respect. For instance, in our country NMDs are supplied with radiopharmaceuticals from various ways. One way is from their own hospital radiopharmacy, which generally is integrated into the NMD and whose staff is also from the same NMD. Another way is from an external or centralized radiopharmacy, which generally supplies doses of radiopharmaceuticals to several NMDs of different hospitals. And other ways can be some hybrid mix of the two above. For instance, in our case the radiopharmacy is inside our NMD, but the staff is from an external company, which prepare and supply the doses of all radiopharmaceuticals, and whose work is conducted under the supervision of a radiopharmacy specialist, who is staff of the NMD.

It is convenient to emphasize that hospital radiopharmacies are facilities for small-scale preparation and dispensation of radiopharmaceuticals. Therefore, they do not need to comply with rules or regulations such as those of GAMP V or EudraLex Vol 4, part I, and Annex 11.

A hospital radiopharmacy must have a radiopharmacy specialist responsible, among other things, for the assurance of traceability. So, when I had to assume this responsibility, I found out that our RIS does not include essential data, such as the activity of the radiopharmaceutical administered to the patient for diagnosis or treatment, nor the batch of the radiopharmaceutical. Therefore, our RIS does not have any traceability capability. I also found that I had to deal with a radiopharmacy information system developed by the external company that supplies us with the radiopharmaceuticals. And it turned out that that software has shortcomings that affect correct traceability. Since that software does not communicate in any way with our RIS, the introduction of radiopharmaceutical prescriptions must be performed manually, that is, by means of the keyboard with the subsequent inevitable typos, which seriously hindered the correct traceability.

Facing with this problem, I developed and implemented our own RPIS (Radiolab). And of course, it would be ideal if it were possible the data transfer between our RIS and our RPIS through the standards of HL7 (Health Level Seven International. https://www.hl7.org). But in this respect, I encountered two impediments. Firstly, implementing HL7 is not possible unilaterally. Even if our RPIS was able to send and receive HL7 messages, it is necessary assistance of the IT department of the hospital with the intranet server. And secondly, the other software application, the RIS in this case, must be able as well to send and receive HL7 messages. But when I tried to contact with the technical support service of the hospital and with the developer of the RIS, in both cases I hit a brick wall, because I did not find any interest in collaboration by either of the two parties. Only one thing was for sure, RIS does not contemplate, nor does it seem that it intends to contemplate, the possibility of recording information regarding the activity and the batch of the radiopharmaceutical administered. So, it is impossible to have traceability from this RIS. However, taking advantage of the fact that this RIS can issue an Excel file with data of the daily prescriptions, I designed in our RPIS the capacity of importing the necessary data from the RIS via an intermediate XLS or XLSX file. With those data, the RPIS issues the daily radiopharmacy worksheet with the scheduled prescriptions. Since the activity of the dose is not contemplated by the RIS, our RPIS assigns an activity to each radiopharmaceutical based on the radiopharmaceutical, the study or treatment, and the patient’s age.

**Fig. 4:**
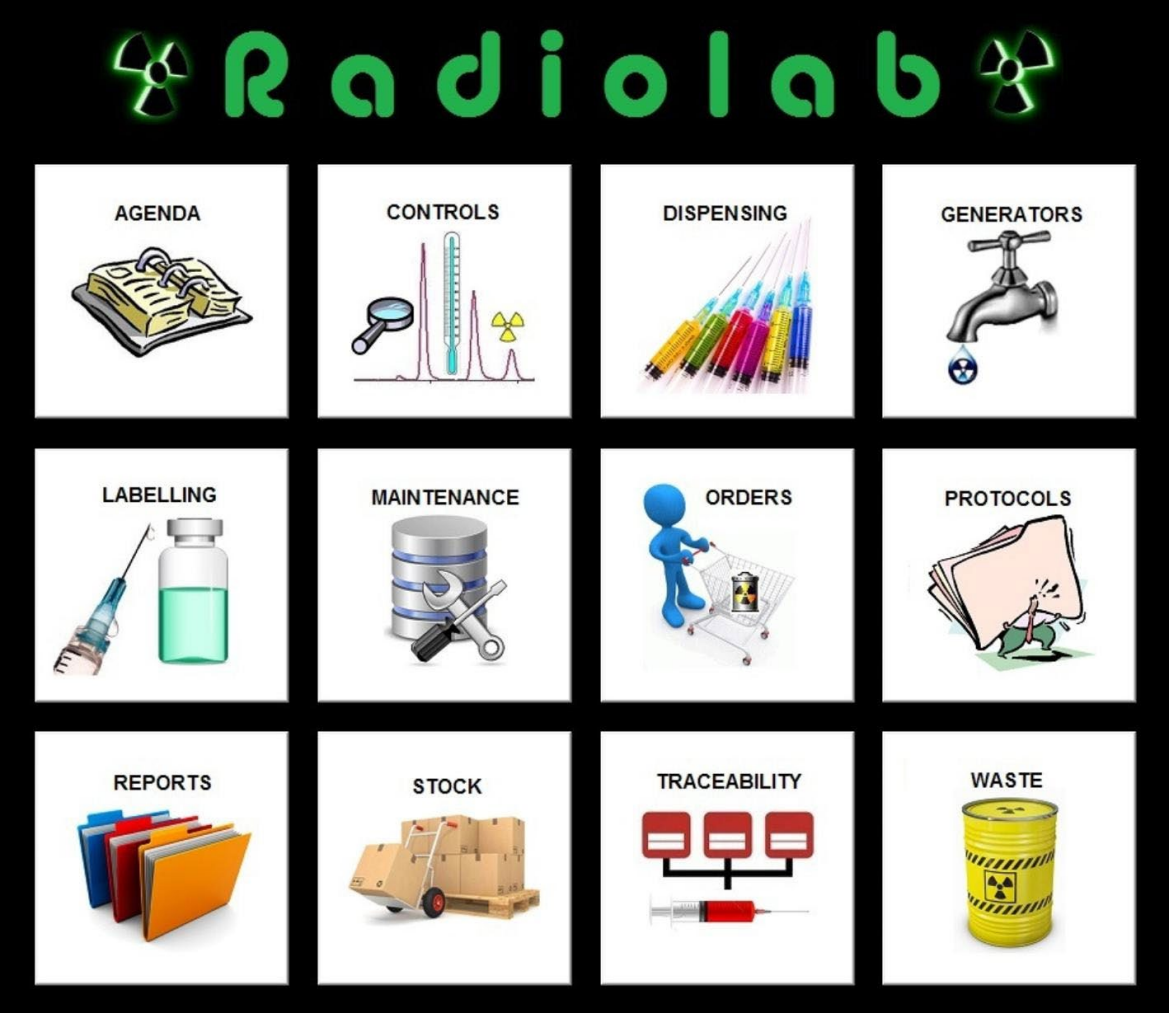
Radiolab main interface

**Fig. 5:**
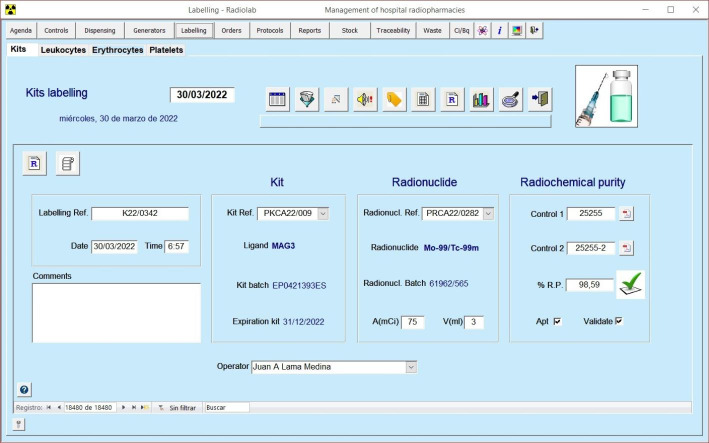
Labelling module interface

Our RPIS is compliant with the standards of good practice established in the guidelines on current good radiopharmacy practice (cGRPP) ( Gillings et al. 2021). It has user-friendly and intuitive interfaces which make it very easy to use (Figs. 4 and 5). And it can work both as a single-user desktop application, and as a multi-user network system, sharing and updating data without overwriting each other’s work.

After installing and before starting to work with this RPIS, it must be customized for each radiopharmacy, entering into the database their data: activimeters, radioactive calibration sources, equipments, staff, catalogue, radionuclides, radioactive products, cold kits, suppliers, protocols, etc. Once all of that has been done, the software is customized and ready to work.

Logically, for as long as we have been using this RPIS its database has been growing. For this reason, it was necessary to foresee a possible need for migrating the MS Access database to SQL Server. But eventually it seems that this is not necessary, and we are going to see why. According the specifications of MS Access, their databases can support up to 255 concurrent users and it supports up to 2 GB in size (https://support.microsoft.com/en-us/office/access-specifications-0cf3c66f-9cf2-4e32-9568-98c1025bb47c).

Regarding the number of concurrent users, if MS Access databases can support up to 255 concurrent users, it means that there will be no problem with 50 concurrent users, that is, five times less than the stated limit. And the staff number of a hospital radiopharmacy, and even of a NMD, is below that figure. And regarding the size of the back-end of the database, the initial size of the just installed back-end file of Radiolab is 2 MB. It was predicted that with the daily work routine of our hospital radiopharmacy, the database would grow around 6 MB per year (Gómez-Perales 2013). Ten years later, the size of our database file was 59 MB. Therefore, that prediction was quite accurate. Therefore, it would take more than 165 years for the database file reaches a size of 1 GB, which is half the size supported by MS Access.

A standard radiopharmacy can generate thousands of printed papers a year (radiochemical quality controls, reports, etc.). A total of 23,144 radiochromatograms have been performed in our radiopharmacy between January 2011 and December 2021 (about 2,100 per year), which represents 46 file cabinets of 500 pages (about 4 file cabinets per year). But with our RPIS there is no need to print the reports of radiochemical purity control or radiochromatograms, since they are stored in digital format (pdf) and they can be located easily from the RPIS at any time. In addition, the validation signature of the control by the radiopharmacy specialists can be done digitally through their user password. Therefore, this software is eco-friendly, because not being necessary to print it saves paper, ink, storage space and time.

A very useful functionality of this RPIS is that all the reports and system data can be converted into common formats (pdf, word, excel), with all the advantages that this entails.

**Fig. 6:**
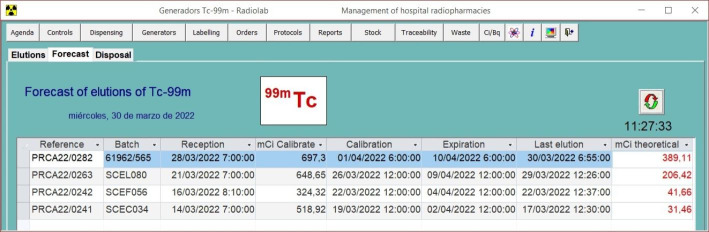
Generator’s module interface: prediction of theoretical activity available on all generators in stock

**Fig. 7:**
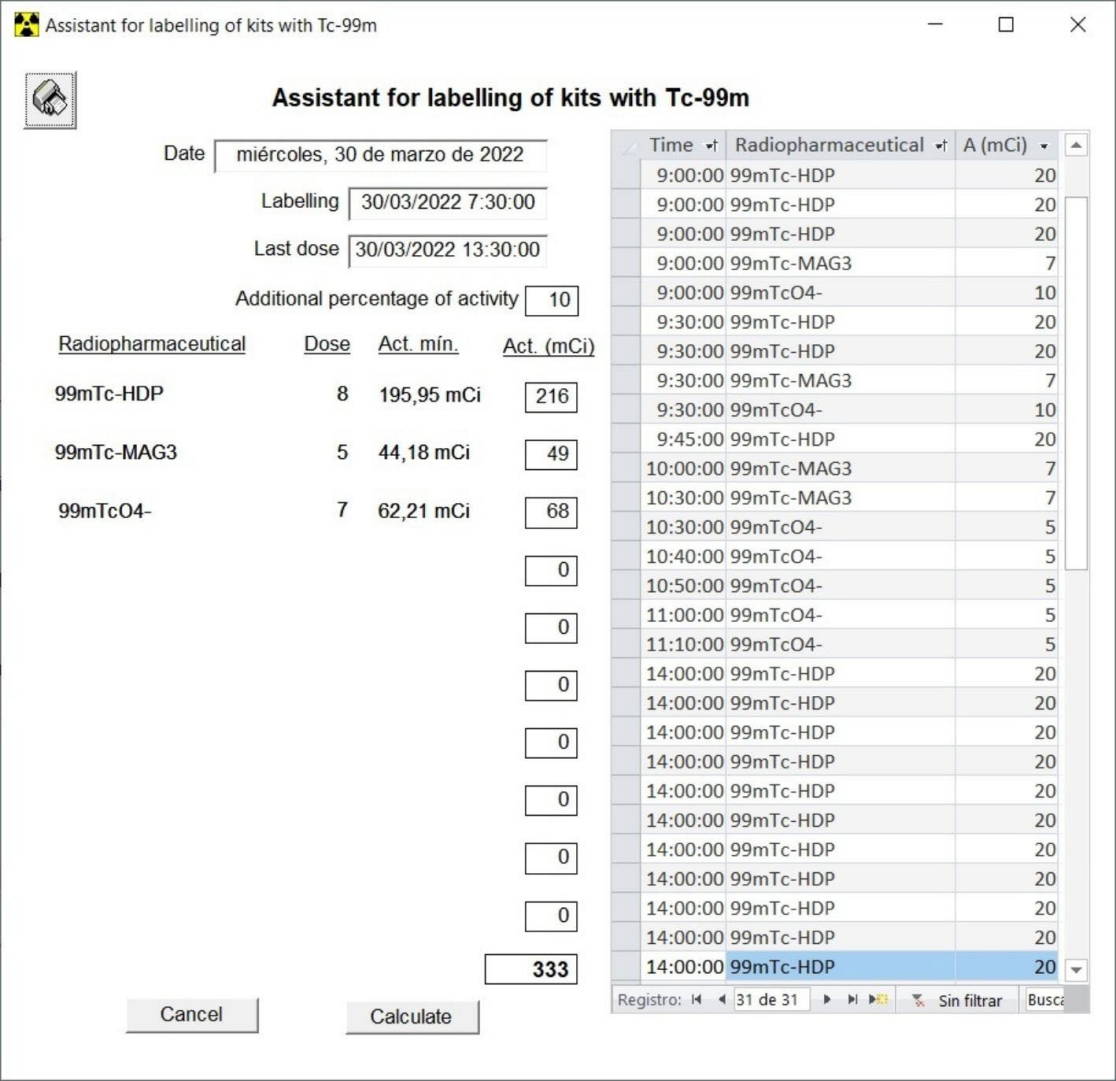
Assistant for labelling of cold kits with 99mTc in the Labelling module

One example of how our RPIS has incorporated new functionalities according to new requirements, is the problem generates form shortages of ^99m^Tc generators supply, which occasionally happens when there is some problem of production or some problem with the transport. In these cases of shortage of ^99m^Tc it is necessary to optimize the use of the elutions of the generators for the labelling of the cold kits. And to do that, two new functionalities have been added to our RPIS: the instant prediction at any time of the theoretical activity available on every generator in the Generators module (Fig. 6), and an assistant for labelling of cold kits with ^99m^Tc in the Labelling module (Fig. 7).

Software validation in hospital pharmacy is generally not formalized. But just recently it has been published a consensual tool for validating radiopharmacy software (Léa et al. 2020), proposed by a panel of experts of the French Society of Radiopharmacy (SoFRa) and following the Delphi method (McPherson et al. 2018). Therefore, to improve our RPIS it has been followed some of the specific requirements of this publication.

A European conformity (CE) marking on any prescription support software is imposed. And, in this regard, it has been published that software in radiopharmacy will eventually acquire medical device status (Blondeel-Gomes et al. 2020). But for now, a RPIS is not a medical software, because it does not comply with any of the conditions mentioned in the European regulation in this regard. A medical software must comply with the definition of a medical device and is considered as such when it fulfils one or more of the functions specified in the Regulation (EU) 2017/745 (https://eur-lex.europa.eu/legal-content/EN/TXT/?uri=CELEX%3A32017R0745). The only software that can carry the CE marking are those that fall into the category of medical device. Software that is not a medical software cannot and must not bear the CE marking.

The benefits provided by Radiolab for the comprehensive management of a hospital radiopharmacy far outweigh the disadvantages it seems to have compared to other commercial applications (laboratory information management systems or LIMS). On one hand, it can be connected to printers and barcode readers; on the other hand, it cannot be connected to other devices, such as dose calibrators, radiochromatography scanners, or dose dispensers. But this last type of connections makes the software much more expensive and less flexible. Besides, are these functionalities essential or even necessary? Is it worth paying a high price to have these features? Sure, that there are several hospital radiopharmacies where they do not want these functionalities, or they cannot afford to pay high prices for them. Additionally, these types of applications require that specialized staff travel to the hospital radiopharmacy, first for its installation and then periodically for its maintenance. And all this makes costs even more expensive. However, Radiolab can be installed and managed by the supervisor in charge of the radiopharmacy, with no need for much computer knowledge. Only in the case of operating through the hospital’s intranet, the collaboration of the hospital’s intranet support team may be necessary.

The flexibility of Radiolab would make possible its application into PET radiopharmacies as it already is or, in any case, by adding some functionality that would be very simple to incorporate.

## Conclusions

A document management system must be in place to provide complete management of hospital radiopharmacy and to ensure correct traceability of radiopharmaceuticals administered in nuclear medicine. Documentation may be paper-based, electronic, or a combination of the two. In the health care area, traditional paper-based file systems have been largely replaced by digital data storage. Therefore, in hospital radiopharmacy management it is about time to make the leap from traditional paper-based file systems into digital data storage. Our radiopharmacy information system Radiolab (www.radiopharmacy.net) has been developed by and for specialists in radiopharmacy. It has been greatly improved during more than ten years since its implementation, and it has proven that it provides comprehensive management and proper traceability in compliance with the cGRPP. It also gives a solution to the problem of data transfer from RIS or NMIS to RPIS when it is still not possible to apply the HL7 standard, or for those hospitals that cannot afford expensive state-of-the-art computer applications for the management of their hospital radiopharmacy and for ensuring traceability.

## Data Availability

The data used in this study are available from the Nuclear Medicine Department of Puerta del Mar University Hospital, but restrictions apply to the availability of these data, which were used under license for the current study, and so are not publicly available. Data are however available from the author upon reasonable request and with permission of the Nuclear Medicine Department of Puerta del Mar University Hospital.

## Abbreviations

cGRPP: current good radiopharmacy practice.
HIS: hospital information system.
ICRP: International Commission on Radiological Protection.
NMD: nuclear medicine department.
NMIS: nuclear medicine information system.
RIS: radiological information system.
RPIS: radiopharmacy information system.
PACS: picture archiving and communication system.
SoFRa: French Society of Radiopharmacy.
